# Is the Scope of Costs Considered in Budget Impact Analyses for Anticancer Drugs Rational? A Systematic Review and Comparative Study

**DOI:** 10.3389/fpubh.2021.777199

**Published:** 2021-11-05

**Authors:** Yue Ma, Yuxin Li, Aixia Ma, Hongchao Li

**Affiliations:** ^1^School of International Pharmaceutical Business, China Pharmaceutical University, Nanjing, China; ^2^Center for Pharmacoeconomics and Outcomes Research, China Pharmaceutical University, Nanjing, China

**Keywords:** budget impact analysis, anticancer drugs, economic evaluation, reimbursement, scope of costs

## Abstract

**Background:** With the increasing disease burden of cancer worldwide, more and more anticancer drugs have been approved in many countries, and the results of budget impact analyses (BIAs) have become important evidence for related reimbursement decisions.

**Objectives:** We systematically reviewed whether BIAs for anticancer drugs consider the scope of costs rationally and compared the results of different cost scopes to provide suggestions for future analyses and decision-making.

**Methods:** Eligible BIAs published in PubMed, Embase, Web of Science, and the Cochrane Library from 2016 to 2021 were identified based on Preferred Reporting Items for Systematic Reviews and Meta-Analyses (PRISMA) guidelines. We extracted 15 terms from the included studies and analyzed how they considered the scope of costs. In addition, a budget impact model was developed for the introduction of geptanolimab to China's National Reimbursement Drug List to enable a comparison of two cost-scope scenarios.

**Results:** A total of 29 studies were included in the systematic review. All 29 studies considered the costs of anticancer drugs, and 25 (86%) also considered condition-related costs, but only 11 (38%) considered subsequent treatment costs. In the comparative study, the predicted budget impacts from 2022 to 2024 were significantly impacted by subsequent treatment costs, with annual differences between the two cost-scope scenarios of $39,546,664, $65,866,161, and $86,577,386, respectively.

**Conclusions:** The scope of costs considered in some existing BIAs for anticancer drugs are not rational. The variations between different cost scopes in terms of budget impact were significant. Thus, BIAs for anticancer drugs should consider a rational scope of costs that adheres to BIA guidelines. Researchers and decision-makers should pay more attention to the scope of costs to achieve better-quality BIAs for anticancer drugs and enhance reimbursement decision-making.

## Introduction

Cancer comprises a large group of diseases involving abnormal cell growth with the potential to invade or spread to other parts of the body uncontrollably (1). Given increasing life expectancy and changes in people's lifestyles, the global disease burden from cancer has gradually risen in recent years. The World Health Organization has identified cancer as the second leading cause of death globally, accounting for an estimated 9.6 million deaths in 2018. The most common forms of cancer are lung, breast, prostate, colorectal, and stomach cancer (2).

To prolong the lives of cancer patients, an increasing number of anticancer drugs have been authorized in many countries. For example, the United States Food and Drug Administration approved 17, 12, and 50 novel anticancer drugs in 2018, 2019, and 2020, respectively (3), while China's National Medical Products Administration authorized more than 30 anticancer drugs for use in relation to various indications in 2020 (4). Although most of these new drugs, including immune checkpoint inhibitors, antibody-drug conjugates, and gene therapies deliver better treatment effects than traditional anticancer drugs, they are more expensive (5–8). Therefore, whether to list these anticancer drugs for national or commercial reimbursement has become an important question for decision-makers.

Budget impact analysis (BIA), which supplements cost-effectiveness analysis (CEA), is a decision-making tool that can be used to predict the financial impact on reimbursement funds of the adoption of a new healthcare technology in a specific healthcare setting. The result of a BIA is generally used to determine the affordability of a new intervention for a specific payer (9, 10). Many countries, such as England, Canada, Australia, and China, have used BIAs to support reimbursement decision-making (11).

The BIA framework generally uses a simple cost-calculator approach that synthesizes costs and epidemiology parameters (12, 13). Regarding BIAs for anticancer drugs, although their accuracy and reliability depend on numerous factors, the scope of costs is crucial. Most cancer patients undergo complex treatment procedures involving the consumption of various medical resources such as drugs, testing, monitoring, and subsequent treatment (i.e., changes in the treatment regimen when the disease progresses) (14). If the costs of all medical resources are met by the same payer, considering different cost scopes will produce different BIA results, sometimes even shifting from cost increases to cost savings.

Some organizations, such as the International Society for Pharmacoeconomics and Outcomes Research (ISPOR), have published BIA guidelines in an effort to standardize the BIA calculation framework, and have provided advice on the scope of costs that need to be considered (15, 16). However, to the best of our knowledge, there have been no studies of the scope of costs used in BIAs for anticancer drugs, with related reviews only focusing on the methodology and providing only a brief summary of the scope of costs (17). Therefore, the scope of costs that BIAs for anticancer drugs have considered, and thus whether they have been rational, are unknown. Given that the scope of costs is crucial, this research gap needs to be addressed.

In this study, we systematically reviewed a range of published BIAs for anticancer drugs, focusing on the scope of costs, and then compared the results of different cost scopes using an example. Our aim was to confirm the necessity of rationally considering the scope of costs used in BIAs for anticancer drugs and to provide guidance for BIAs and relevant decision-makers.

## Methods

### Existing Recommendations

Some guidelines for BIAs have been published in an effort to standardize research procedures. To obtain a better understanding of existing recommendations regarding the scope of costs used in BIAs, we searched for and summarized these guidelines. Based on a previous review (18), 10 BIA guidelines were reviewed. These had been published by various organizations and countries including the ISPOR, the National Institute for Health and Clinical Excellence (NICE) in the United Kingdom, Canada, France, Ireland, Australia, the Netherlands, Belgium, Thailand, and Poland (16, 19–27). Microsoft Excel 2016 was used to summarize the various recommendations.

### Systematic Review

This systematic review was conducted and reported in accordance with the Preferred Reporting Items for Systematic Reviews and Meta-Analyses (PRISMA) guidelines (28). PubMed, EMBASE, Web of Science Core Collection, and the Cochrane Library were searched for studies published on BIAs for anticancer drugs from 1 January 2016 to 26 July 2021. The search terms and the detailed search strategies are presented in Appendix 1.

Studies were included if they (1) were original BIAs pertaining to anticancer drugs used to treat cancer patients, (2) reported the scope of costs, and (3) were published in English. Studies were excluded if they (1) did not focus on cancer patients, (2) did not include the scope of costs, (3) calculated the budget impact of biosimilars compared with that of the original drug (biosimilars usually have similar effects at a lower price compared with the original drugs, and thus a comprehensive scope of costs is generally considered unnecessary), or (4) were published in the form of a systematic review, meta-analysis, abstract, or dissertation. The literature search and screening were undertaken independently by two investigators. Any disagreements were adjudicated by senior investigators.

On the basis of the ISPOR Task Force guidelines (15, 16), we developed an evidence table summarizing how each study was designed, the scope of costs considered, and the results. The scope of costs was divided into two parts. The first part included the costs of intervention (target anticancer drugs), and was calculated by multiplying the unit price of the anticancer drug by the amount used in the target population. The second part included the impact on other costs, which consisted of two components: condition-related costs and indirect costs. In BIAs, condition-related costs usually include monitoring costs (costs of medical resources about monitoring disease progression or other events, e.g., imaging examination, laboratory examination and blood pressure monitoring), administration costs (costs of acquiring and using drugs, e.g., drug preservation and injection), adverse event (AE) costs (costs of drugs for AE management, e.g., leucocyte increasing agent for leukopenia), and subsequent treatment costs (costs of treatment after disease progression, e.g., immunotherapy after chemotherapy failure), while indirect costs usually include the costs of lost productivity and social services, referring to the working hours and productivity loss due to disease, disability, or death which includes the loss of salary for patients and their families/caregivers caused by discontinuing school, sick leave, and early death, etc., and in most cases are only considered when adopting the societal perspective. In addition to the scope of costs, 14 items regarded as essential for BIAs were included in the evidence table (29, 30): country, intervention, research funding, perspective, supported decision-making, target population, time horizon, market share, comparator(s), treatment duration, results of the BIA, uncertainty and scenario analyses, validation, and data sources. Then, we systematically extracted data and summarized the scope of costs considered in all of the included studies in evidence tables using Microsoft Excel 2016.

### Comparative Study

To illustrate the influence of the scope of costs on BIA results, we developed a Microsoft Excel-based budget impact model for an anticancer drug and estimated two cost-scope scenarios: scenario 1, which did not consider subsequent treatment costs, and scenario 2, which considered subsequent treatment costs. We then compared the results of these two cost-scope scenarios. The model was developed based on ISPOR guidelines (15, 16).

**Figure 1:**
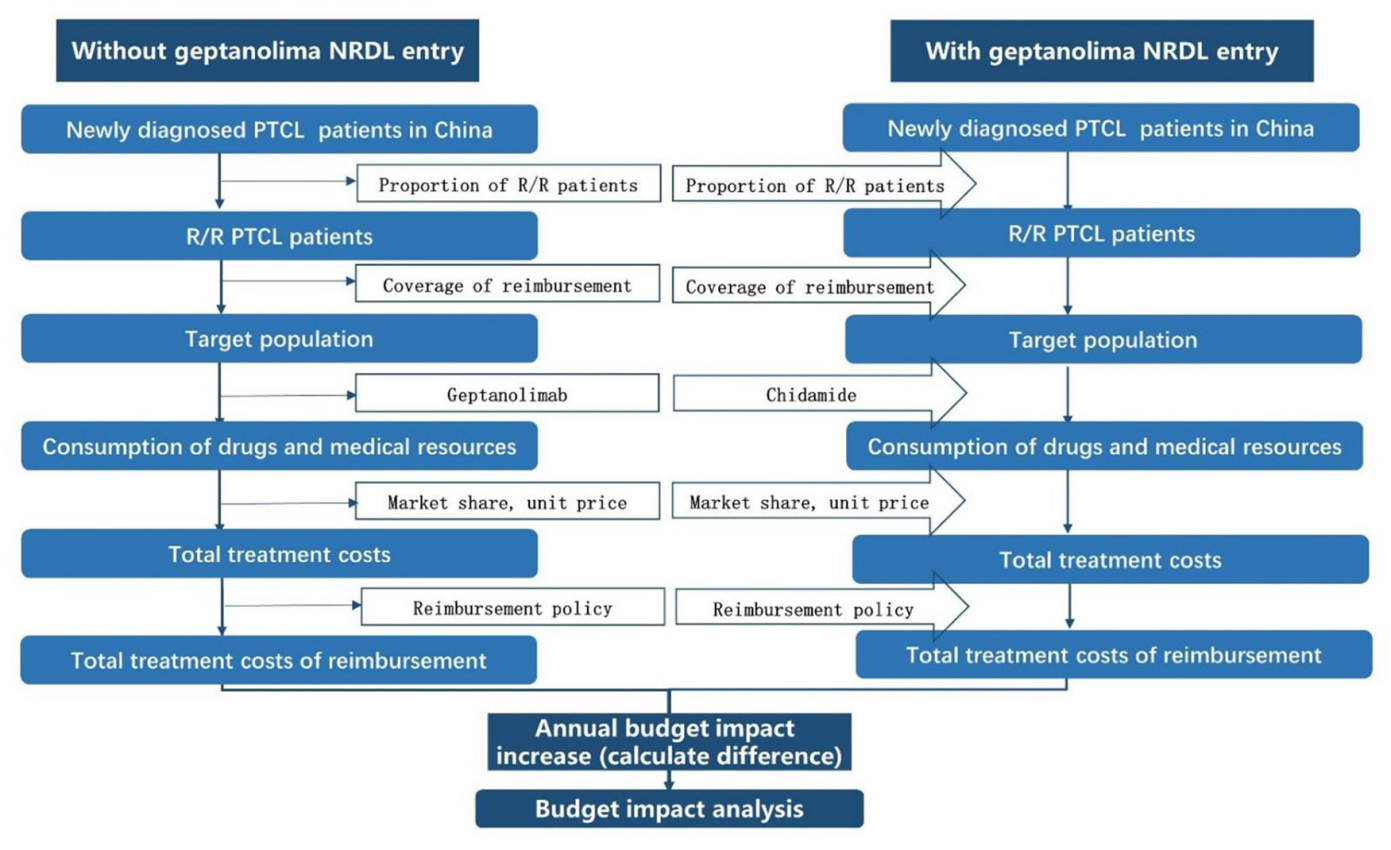
Model structure. NRDL, National Reimbursement Drug List; R/R PTCL, relapsed or refractory peripheral T cell lymphoma.

This model was built to estimate the budget impact of introducing geptanolimab as a treatment option for patients with relapsed or refractory peripheral T cell lymphoma (R/R PTCL) from the perspective of China's National Healthcare Security Administration. The target population was the annual number of new patients with R/R PTCL. The model conceptualized two distinct market scenarios: (1) a status quo scenario in which geptanolimab was not included in the National Reimbursement Drug List (NRDL), which only included chidamide for the treatment of R/R PTCL; and (2) an alternative scenario in which geptanolimab was included in the NRDL and offered as an alternative treatment to chidamide. The budget impact was the cost difference between the two market scenarios. The baseline year was 2021 and the time horizon was 3 years. The structure of the model is shown in Figure 1.

Demographic and epidemiological data were obtained from published studies, statistical yearbooks, and expert interviews (31–34).

As chidamide was the only drug listed in the NRDL for treatment of R/R PTCL, we assumed that the market share for chidamide was 100% in the scenario without geptanolimab, and in the scenario in which geptanolimab was included in the NRDL, we assumed that geptanolimab would gradually replace chidamide over time. The various market shares were based on available sales data for chidamide (35).

Based on the study perspective, in scenario 1 we only considered drug costs and condition-related costs, which included drug acquisition costs, inspection and testing costs, disease management costs, and AE management costs. As mentioned above, in scenario 2, we also considered subsequent treatment costs. All interventions and condition-related costs and reimbursement-related parameters were based on the Menet Medical Information database, published literature, China's National Healthcare Security Administration website, expert interviews, and assumptions (36–39). All costs were expressed in 2021 US dollars (USD) using the prevailing exchange rate of 1 Chinese yuan (CNY) = 0.1547 USD (40).

In addition, we assumed that the median progression-free survival rate was equal to the average treatment duration using geptanolimab and chidamide. We also used the rate of disease progression in 1 year under treatment with geptanolimab and chidamide to estimate the number of patients who were likely to experience further progression and require further treatment each year. Clinical efficacy data for geptanolimab and chidamide were obtained from published clinical trials (38, 39). Subsequent treatment regimens and the proportions of cancer patients who received each subsequent treatment regimen were obtained from clinical guidelines and expert interviews (41).

A summary of all model inputs and their sources is presented in Table 1, and detailed information is presented in Appendix 2.

**Table 1:** Model inputs of budget impact model.

	**Parameters**	**Values**	**Sources**
Total population of China			
	Year 1 (2022)	1,418,677,976	(31)
	Year 2 (2023)	1,424,885,933	
	Year 3 (2024)	1,431,093,889	
Epidemiological parameters			
	Incidence of NHL	0.00429%	(32)
	The proportion of PTCL in NHL	21.40%	(33)
	Incidence of PTCL in 2020	0.00092%	Calculation
	Compound annual growth rate of PTCL incidence	3%	(34)
	Proportion of patients with R/R PTCL	75%	Assumption
	Visiting rate of patients with R/R PTCL	100%	Assumption
	Proportion of patients with R/R PTCL receiving treatment	100%	Expert interview
	Adherence of patients with R/R PTCL receiving treatment	100%	
	Hospitalization days per month for PFS patients	3	
	Outpatient days per month for PFS patients	27	
	Proportion of patients receiving subsequent treatment	100%	
Reimbursement parameters of Basic Medical Insurance			
	Coverage rate	100%	(36)
	Outpatient reimbursement ratio	50%	
	Hospitalization reimbursement ratio	65%	
	Proportion of costs covered other than drugs	70%	Assumption
Treatment duration parameters (m)			
	Average treatment duration of geptanolima (median PFS)	3.7	(38)
	Proportion of disease progression patients previous received geptanolima	82.93%	
	Average treatment duration of chidamide (median PFS)	2.1	(39)
	Proportion of disease progression patients previous received chidamide	89.38%	
Cost parameters ($)			
	Annual average treatment costs of geptanolima	7,425.60	(37–39), Expert interview, Assumption
	Annual average treatment costs of chidamide	7,004.20	
	Annual average inspection and testing costs of geptanolima	2,498.75	
	Annual average inspection and testing costs of chidamide	1,688.34	
	Annual average disease management costs of geptanolima	305.66	
	Annual average disease management costs of chidamide	206.52	
	Annual average AE management costs of geptanolima	2.74	
	Annual average AE management costs of chidamide	0.80	
	Annual average subsequent treatment costs of geptanolima	59,847.20	
	Annual average subsequent treatment costs of chidamide	72,031.25	

*NHL, non-Hodgkin's lymphoma; PFS, progression free survival; R/R PTCL, relapsed or refractory peripheral T cell lymphoma*.

## Results

### Existing Recommendations

The study perspective is related to what resources should be assessed in BIA. The majority of the guidelines included recommend that the perspective should be that of the healthcare payer or the budget holder (16, 19, 21–27). However, the choice of perspective might differ under some specific circumstances. For example, The Netherlands recommends a wider societal perspective when the healthcare payer is the government (24). For Canada, the guideline recommends the assessment of the budget impact related to drugs only with public purchaser perspective (20). Regarding time horizon, all the guidelines examined recommend that it depends on the duration of the budgeting period, target intervention diffusion rate and type of intervention and condition (16, 20–27). The guidelines of ISPOR and Thailand recommends 1–5 years of time horizon (16, 26), of Canada and Poland recommend 2–3 years (20, 27), of French suggests 3–5 years (21), of Australia suggests over 6 years (23), of Belgium recommends a minimum of 3 years (25) and NICE recommends 5 years (19).

The existing recommendations regarding the scope of costs are summarized in Table 2. Regarding direct costs, all 10 guidelines examined recommend that intervention costs, AE management costs, and subsequent treatment costs should be considered in BIAs (16, 19–27), and eight of the 10 guidelines recommend that administration costs and monitoring costs should also be considered (16, 19, 21, 22, 24–27). Regarding indirect costs, none of guidelines recommend to consider them routinely in BIAs, and only four guidelines recommend that they should be considered in specific circumstances whereby indirect costs significantly influence the results or are able to be reasonably estimated (16, 21, 25, 27). For example, the guideline of Belgium suggests that indirect costs should not be included in a BIA as these are not generally relevant to the budget holder, however they can be included in a BIA as a complementary analysis if they are significant (25).

**Table 2:** Summary of key recommendations of cost scopes in existing BIA guidelines.

**BIA guidelines**			**ISPOR**	**NICE**	**Canada**	**France**	**Ireland**	**Australia**	**The Netherlands**	**Belgium**	**Thailand**	**Poland**
			**(2014)**	**(2017)**	**(2020)**	**(2018)**	**(2018)**	**(2006)**	**(2016)**	**(2014)**	**(2014)**	**(2004)**
Recommended scope of costs	Direct costs	Intervention costs	√	√	√	√	√	√	√	√	√	√
		Administration costs	√	√		√	√		√	√	√	√
		Monitoring costs	√	√		√	√		√	√	√	√
		AE costs	√	√	√	√	√	√	√	√	√	√
		Subsequent treatment costs	√	√	√	√	√	√	√	√	√ (Depending on the payer requirement and perspectives)	√
	Indirect costs		√ (When needed)			√ (Depending on perspectives)				√ (When needed)		√ (If possible to predict)

*AE, Adverse events; BIA, Budget impact analysis; ISPOR, International Society for Pharmacoeconomics and Outcomes Research; NICE, National Institute for Health and Clinical Excellence of United Kingdom; √, Recommended to consider*.

### Systematic Review

**Figure 2:**
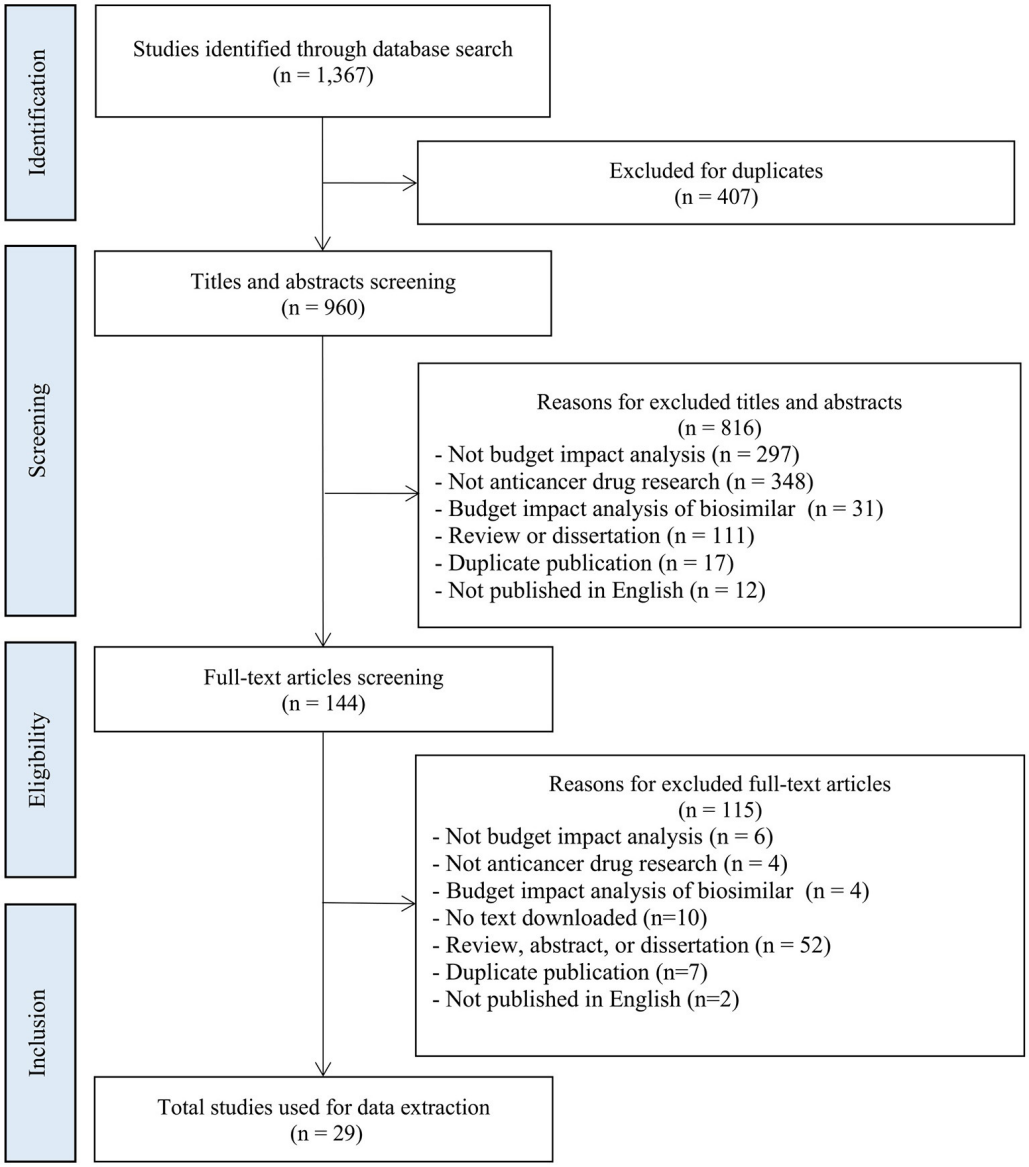
Flow diagram of literature search and study identification.

A total of 1,367 articles were initially identified, of which 29 articles were included in the final analysis (42–70). Figure 2 shows a flowchart of the literature screening process.

The characteristics of the included studies are summarized in Table 3 and Appendix 3. Most of the included studies originated in the United States (*n* = 18, 62%) (42–46, 48, 50–59, 64, 66), followed by Italy (*n* = 2, 7%) (69, 70) and one from each of Brazil (60), the Netherlands (62), France (47), Japan (49), Norway (65), Saudi Arabia (61), Spain (63), and Thailand (67), as well as one multi-country study (58). The studies covered 11 types of cancer including non-small-cell lung cancer (*n* =8, 28%) (43, 45, 47, 54, 55, 57, 62, 65), prostate cancer (*n* =5, 17%) (42, 44, 48, 59, 64), colorectal cancer (*n* = 4, 14%) (60, 67, 68, 70), ovarian cancer (*n* = 3, 10%) (46, 53, 63), breast cancer (*n* = 2, 7%) (56, 61), myeloma (*n* = 2, 7%) (49, 58), melanoma (*n* = 1, 3%) (52), head and neck cancer (*n* = 1, 3%) (69), cell carcinoma of the urothelium (*n* = 1, 3%) (50), gastroenteropancreatic neuroendocrine tumor (*n* = 1, 3%) (66), and epithelial ovarian, fallopian tube or primary peritoneal cancer (*n* = 1, 3%) (51). Most of the interventions in these studies involved innovative anticancer drugs, including selective poly ADP-ribose polymerase (PARP)-1 and PARP-2 inhibitor (e.g., niraparib) (51, 53), epidermal growth factor receptor (EGFR) and human epidermal growth factor receptor 2 tyrosine kinases inhibitor (e.g., afatinib) (55, 62), immune checkpoint inhibitors (e.g., nivolumab, pembrolizumab) (47), and vascular endothelial growth factor inhibitor (e.g., bevacizumab) (60).

**Table 3:** Characteristics of included studies.

**Author**	**Year**	**Country**	**Intervention**	**Research foundation**	**Perspective**	**Supported decision-making**	**Target population**	**Time horizon**	**Market share**	**Comparator(s)**	**Treatment duration**	**Scope of costs**			
												**Costs of intervention**	**Condition-related costs**	**Indirect costs**	**Considering subsequent therapy**
Appukkuttan (42)	2020	US	Darolutamide+ADT	Model	The third-party payer	Insurance coverage	Adult males with nmCRPC	5 years	Substitution	Apalutamide + ADT, Enzalutamide + ADT, Branded AA + Prednisone + ADT, Generic AA + Prednisone + ADT, ADT Alone.	Based on approved use of drugs	Drug cost	Administration (physician visits; injection); AE management	/	No
Cai (43)	2020	US	Capmatinib	Model	Commercial and Medicare payer	Insurance coverage	Patients with mNSCLC with METex14 skipping mutations	3 years	Substitution	Crizotinib, Pembrolizumab, Nivolumab, Docetaxel, Pemetrexed, Gemcitabine, Ramucirumab + Docetaxel, Carboplatin + Pemetrexed, Carboplatin/Cisplatin +Pemetrexed/Paclitaxel, Pembrolizumab + Carboplatin + Paclitaxel/Nab-Paclitaxel, Pembrolizumab + Carboplatin/Cisplatin +Pemetrexed, Best Supportive Care.	Median treatment duration, median PFS as a proxy	Drug cost	Administration; Medical (pre-progression, AE management, progression, terminal care, and monitoring services); Testing (NGS)	/	Yes
Mason (44)	2021	US	Adaptive abiraterone therapy	Clinical study	CMS	Insurance coverage	Patients with metastatic CRPC	/	Substitution	Standard Continuous Abiraterone Therapy	Days received therapy	Drug cost	The costs of care beginning with the first dose until treatment stopped	/	No
Stargardter (45)	2021	US	Tepotinib	Model	Health plan	Insurance coverage	Adult patients with mNSCLC harboring METex14 skipping alterations	3 years	Substitution	Capmatinib, Crizotinib, SOC	Median time on treatment, median PFS as a proxy	Drug acquisition and administration costs	Monitoring, disease and AE management, subsequent treatment, biomarker testing	/	Yes
Wallace (46)	2020	US	Rucaparib	Model	Health plan	Insurance coverage	Patients with Metastatic Ovarian Cancer	3 years	Substitution	Maintenance therapy: Rucaparib, Olaparib, Niraparib, Bevacizumab. Treatment cohort: Olaparib, Bevacizumab + CT, other CT.	Median treatment duration	Drug acquisition and administration costs	Monitoring, AE management, Post-drug BSC	/	Yes
Monirul (47)	2020	France	Nivolumab+ Pembrolizumab	Model	Public health insurance	Insurance coverage	Patients treated for metastatic 1st line or 2nd line NSCLC	1 year	Substitution	/	Number of cycles received per year by patients	Drug cost	/	/	No
Schultz (48)	2020	US	Enzalutamide	Model	Health plan	Insurance coverage	Newly incident patients with high-risk nmCRPC	3 years	Substitution	Apalutamide + ADT, Bicalutamide + ADT, ADT Only	Average MFS	Drug acquisition and administration costs	Monitoring, AE management, medical visit, disease progression	/	No
Yamazaki (49)	2020	Japan	Nilotinib	Model	Public payer	Insurance coverage	Patients eligible for TFR	3 years	Substitution	TFR	Continuation of treatment until disease progression	Drug acquisition and administration costs	Hospital/ physician visits, molecular monitoring	/	No
Kongnakorn (50)	2019	US	Avelumab	Model	Commercial and Medicare payer	Insurance coverage	Patients with locally advanced or metastatic TCCU	3 years	Substitution	Atezolizumab, Durvalumab, Nivolumab, Pembrolizumab, CT	Median TTF	Drug cost	Administration, AE (grade ≥3) management and related HRU, post-progression (BSC)	/	Yes
Neeser (51)	2019	US	Niraparib	Model	US payers	Insurance coverage	Adult patients with recurrent epithelial ovarian, fallopian tube or primary peritoneal cancer	3 years	Substitution	Olaparib, Rucaparib, Bevacizumab, W&W	A quarter	Drug costs	Monitoring, AE management, subsequent treatment	/	Yes
Stellato (52)	2019	US	Dabrafenib + Trametinib	Model	Commercial Payer	Insurance coverage	Patients with resected Stage IIIA-C melanoma and BRAF mutation-positive	3 years	Substitution	Observation, High-Dose Interferon Alfa-2B, Ipilimumab, Nivolumab	A 6-month cycle duration	Drug acquisition and administration costs	Monitoring, AE management, subsequent treatment, BRAF testing, terminal care	/	Yes
Wu (53)	2019	US	Niraparib + Olaparib	Model	The third-party payer	Insurance coverage	Patients with platinum-sensitive, recurrent ovarian cancer	1 year	Substitution	Bevacizumab, Rucaparib	Median PFS	Drug acquisition and administration costs	Monitoring, AE management	/	No
Bly (54)	2018	US	Necitumumab	Model	Commercial and Medicare payer	Insurance coverage	MsqNSCLC patients eligible to receive first-line CT	3 years	Substitution	Gemcitabine & Cisplatin, Gemcitabine & Carboplatin, Paclitaxel & Carboplatin, Nab-Paclitaxel & Carboplatin.	Treatment duration in clinical trials.	Drug acquisition and administration costs	AE management, diagnosis, comorbidities, posttreatment care, hospice care	/	Yes
Graham (55)	2018	US	Afatinib	Model	Health plan	Insurance coverage	Adult patients with mNSCLC having EGFR del19 or L858R mutations initiating first-line treatment	5 years	Substitution	Afatinib, Erlotinib, Gefitinib, Pemetrexed/Cisplatin.	Continuation until disease progression	Drug acquisition and administration costs	AE management, progressive disease costs (continuing-care costs and end-of-life costs)	/	Yes
Mistry (56)	2018	US	Ribociclib + Letrozole	Model	The third payer	Insurance coverage	Postmenopausal women with HR+/HER2- advanced or metastatic breast cancer	3 years	Substitution	Palbociclib + Letrozole	Median PFS	Drug acquisition and administration costs	Treatment administration, health state management, or disease management, outpatient visits, bone metastases management, hospitalization, laboratory testing, imaging or palliative care (for subsequent treatment only), monitoring, AE management	/	Yes
Goldstein (57)	2017	US	Pembrolizumab	Model	Society	Avoidance of drug wastage	Patients with PD-L1-positive mNSCLC treated with pembrolizumab annually in the first-line setting	1 year	Substitution	Fixed dosing of Pembrolizumab	A maximum of 2 years (35 cycles) or until disease progression	Drug cost	/	/	No
Bloudek (58)	2016	US	Panobinostat	Model	Commercial and Medicare payer	Insurance coverage	Adult patients initiating salvage therapy for RRMM	1 year	Substitution	Bortezomib-Dexamethasone, Lenalidomide-Dexamethasone, Lenalidomide-Bortezomib-Dexamethasone, Carfilzomib Monotherapy, Carfilzomib-Lenalidomide-Dexamethasone, Pomalidomide-Dexamethasone.	Median DOT reported in product labeling or clinical trials.	Drug cost	Administration, AE management, monitoring	/	No
Bui (59)	2016	US	Enzalutamide	Model	The third-party payer	Insurance coverage	CT-naïve adult patients with mCRPC	1 year	Substitution	Abiraterone Acetate, Sipuleucel-T, Radium Ra 223 Dichloride, Docetaxel.	Prescribing time	Drug cost	Administration, subsequent treatment, monitoring, AE management	/	No
Silva (60)	2021	Brazil	Bevacizumab, Cetuximab, Panitumumab.	Model	Unified Health System	Future decision making of Unified Health System in Brazil	Patients with CT-refractory mCRC	5 years	Substitution	CT	Reimbursement value records	Drug cost	/	/	No
Elsamany (61)	2021	Saudi Arabia	Trastuzumab	Model	Governmental health sector	Insurance coverage	Adult patients with early and metastatic HER2-positive breast cancer	3 years	Substitution	Trastuzumab	17 cycles (3 weeks per cycle)	Drug cost	Administration	/	No
Westerink (62)	2020	Dutch	Afatinib	Model	Healthcare system	Insurance coverage	Patients with mNSCLC having EGFR deletion 19 or L858R mutations initiating first-line treatment.	5 years	Substitution	Osimertinib	Median PFS	Drug cost	AE management, mutation testing, subsequent treatment	/	Yes
Delgado-Ortega (63)	2018	Spain	Olaparib	Model	National Health System	Insurance coverage	Patients with BRCA-mutation positive, PSR HGSOC	5 years	Substitution	W&W, Bevacizumab	Continuation until disease progression	Drug cost	Administration, AE management, BRCA gene testing, subsequent treatment	/	Yes
Flannery (64)	2017	US	Cabazitaxel	Model	Heath plan	Insurance coverage	Patients with mCRPC progressing after treatment with docetaxel	1 year	Substitution	Abiraterone Acetate, Enzalutamide, Radium-223.	Prescribing time	Drug cost	administration, AE management	/	No
Norum (65)	2017	Norway	Pembrolizumab	Model	Regional Health Authority	Hospitals' budgets	Patients with NSCLC being PD-L1 positive in second-line therapy	1 year	Substitution	Docetaxel, Pemetrexed, Navelbine, Erlotinib, Gefitinib.	Mean number of treatment cycles.	Drug costs	PD-L1 testing, Radiology (CT, MR), Pulmonologist/ oncologist/nurse, pharmacy and traveling expenses	/	No
Ortendahl (66)	2017	US	Lanreotide Or Octreotide	Model	Hospital	Hospitals' budgets	Patients with GEP-NETs	1 year	Substitution	Lanreotide+ Octreotide	Calculating average cost per treated patient in hospital database directly	Drug acquisition and administration costs	/	/	No
Kulthana chairojana (67)	2020	Thailand	HC	Model & Clinical study	Society	Service reimbursement	Patients with stage III CRC	1 year	Substitution	IP	12 cycles (6 months) following the guidelines	Drug acquisition and administration costs	Healthcare personnel, laboratory tests, surgical procedure for central line, AE management, equipment, home health services	Nursing time, dispensing fees	No
Hanna (68)	2021	Australia, Denmark, New Zealand, Spain, Sweden and the UK	Fluoro pyrimidine-Oxaliplatin	Clinical study	Countries recruited to SCOT	Insurance coverage	Patients diagnosed with stage II or III CRC	5 years	Substitution	Adjuvant, Fluoropyrimidine-Oxaliplatin CT.	3 months	Drug acquisition and administration costs	Treatment, hospitalizations	/	No
Mennini (69)	2019	Italy	Cetuximab	Model	Society	Insurance coverage	Patients with RM HNSCC	2 months	Substitution	Cetuximab	Median PFS	Drug cost	Medical examination/ administration (physician, nurse, consumption material, drug administration, hospital pharmacy)	Working day Italy (the loss of productivity or absence from work of the patient or caregiver)	No
Mennini (70)	2019	Italy	Cetuximab	Model	National health system	Insurance coverage	Patients with mCRC RAS wild-type	10 months	Substitution	Cetuximab	Duration of the first-line treatment	Drug cost	Cost of medical examination per administration (including the cost of the physician, nurse, consumption material, for the drug administration and distribution by the hospital pharmacy)	Working day Italy (the loss of productivity or absence from work of the patient or caregiver)	No

*AA, abiraterone acetate; ADT, androgen deprivation therapy; AE, Adverse events; BRAF, v-Raf murine sarcoma viral oncogene homolog B; BSC, Best supportive care; CRC, Colorectal cancer; CMS, Centers for Medicare & Medicaid Services; CT, Chemotherapy; CDK 4/6, Cyclin-dependent kinase 4 and 6; CRPC, Castration-resistant prostate cancer; DOT, Duration of treatment; EGFR, Epidermal growth factor receptor; GEP-NETs, Gastroenteropancreatic neuroendocrine tumors; HR+, Hormone receptor-positive; HRU, Healthcare resource utilization; HER2–, Human epidermal growth factor receptor 2-negative; HNSCC, Head and neck squamous cell cancer; HGSOC, High-grade serous ovarian cancer; HC, Home-Based chemotherapy; IP, Hospital-based chemotherapy treatment; mCRC, Metastatic colorectal cancer; METex14, Mesenchymal–epithelial transition exon 14; MsqNSCLC, Metastatic Squamous Non-Small Cell Lung Cancer; mNSCLC, Metastatic non-small cell lung cancer; MR, Magnetic resonance; MFS, Metastasis-free survival; nmCRPC, non-metastatic castration-resistant prostate cancer; NSCLC, Non-small cell lung cancer; NGS, Next-generation sequencing; Neci + GCis, Necitumumab + gemcitabine and cisplatin; PFS, Progression-free survival; PD-L1, Programmed death ligand 1; PD-L1, Programmed cell death ligand; PSR, Platinum-sensitive; RRMM, Relapsed and/or refractory multiple myeloma; RM, Recurrent and/or metastatic; SCOT, Short Course Oncology Treatment; SoC, Stand of care; TFR, Treatment-free remission; TKI, Tyrosine kinase inhibitor; TCCU, Transitional cell carcinoma of the urothelium; TTF, Time-to-treatment failure; W&W, Watch And Wait*.

Regarding the perspective, three studies (10%) considered the societal perspective (57, 67, 69), four studies (14%) considered the healthcare system perspective (60, 62, 63, 70), and all other studies (*n* = 22, 76%) considered the budget-holder perspective (42–56, 58, 59, 61, 64–66, 68). Regarding the budget-holder perspective, one study calculated the budget impact from the hospital perspective (66), five adopted the health-plan perspective (45, 46, 48, 55, 64), and 16 adopted the third-party payer perspective including public health insurance, Medicare, and commercial insurance (42–44, 47–54, 56, 58, 59, 61, 65, 68). Five studies reported results from more than one perspective (43, 50, 54, 58, 68).

The focus of our study was the scope of costs included in the BIAs of the sample studies. All of the studies considered the costs of anticancer drugs, most of which were calculated based on the unit prices and treatment duration assumptions. In 12 studies (41%), the authors assumed a treatment duration on the basis of treatment effect data, including the median progression free survival, the average metastasis-free survival, the median time to treatment failure, and the time until relapse (43, 45, 48–50, 53, 55–57, 62, 63, 69). In 10 studies (34%), they assumed a treatment duration based on actual patient treatment durations including days spent receiving therapy, treatment in clinical trials, and prescribed treatment durations (44, 46, 51, 54, 58–60, 64, 68, 70). In six studies (21%), the treatment duration was based on either the drug administration instructions or clinical guidelines (42, 47, 52, 61, 65, 67). Only one study (3%) based the treatment duration on hospital data (66).

Regarding condition-related costs, 25 studies (86%) considered these, most of which included administration costs (e.g., physician visits, injections, consumption of materials, and hospital pharmacy costs), AE management costs (e.g., grade ≥3 AE), gene-testing costs (e.g., BRCA testing and EGFR testing), and hospitalization costs. Of these studies, 12 (41%) provided a clear reason why they considered condition-related costs, while the other 13 (48%) reported condition-related costs directly. For example, Stargardter et al. (45) considered condition-related costs because they adopted ISPOR task force recommendations, while Appukkuttan et al. (42) considered the costs associated with prostate cancer in the United States without providing any reference framework. In addition, although there were 25 studies considering condition-related costs, only 11 (38%) considered subsequent treatment costs. Of those, in five studies (14%), the authors took subsequent treatment costs based on guideline recommendations into account, while in the other six studies (24%), the authors took the subsequent treatment costs based on their own model structure and relative considerations into account. For example, Kongnakorn et al. (50) calculated the costs of post-progression (on subsequent 3 line active treatment) and post-progression/off-treatment (best supportive care) as subsequent treatment costs, while Stellato et al. (52) developed a Markov model and considered the costs of subsequent recurrence events in patients. Bly et al. (54) and Mistry et al. (56) estimated the costs of patients receiving subsequent lines of therapy after the first-line therapy using their target drugs. Graham et al. (55) assumed that progressive disease costs comprised monthly continuing-care costs that were applied each month between progression and the final year of life, and end-of-life costs that were applied each month during the final year of life. Delgado-Ortega et al. (63) considered all treatment lines in their model and assumed that patients received maintenance treatment until disease progression, and then received chemotherapy.

Four studies (14%) did not consider condition-related costs. Monirul et al. (47) and Goldstein et al. (57) assumed that all costs other than for drugs were equivalent under the two strategies, while Silva et al. (60) and Ortendahl et al. (66) did not mention costs other than those for drugs in their studies.

Two studies (7%) considered indirect costs, which included loss of productivity or absence from work of either the patient or a caregiver (69, 70). The main reason why they considered indirect costs was the societal perspective they adopted in their studies.

### Comparative Study

Based on demographic and epidemiological data, it was estimated that there would be 7,885, 9,050, and 10,017 newly diagnosed patients with R/R PTCL in China in 2022, 2023, and 2024, respectively. These patients would receive treatment with either geptanolimab or chidamide. The resulting market shares are shown in Table 4. Based on the target population, market shares, and disease progression data, it was estimated that in the scenario without geptanolimab in the NRDL, the number of chidamide patients receiving subsequent treatment was 7,048, 8,089, and 8,953 in the 3 years, and in the scenario with geptanolimab in the NRDL, the number of geptanolimab patients receiving subsequent treatment was 1,886, 2,384, and 2,831 in the 3 years and the number of chidamide patients receiving subsequent treatment was 5,015, 5,519, and 5,902 in the 3 years.

**Table 4:** Market share inputs.

**Intervention**	**Market share**			**Sources**
**Years**	**2021**	**2022**	**2023**	
Without geptanolima NRDL entry				
Geptanolima	0%	0%	0%	
Chidamide	100%	100%	100%	(25), Assumption
With geptanolima NRDL entry				
Geptanolima	28.84%	31.77%	34.08%	
Chidamide	71.16%	68.23%	65.92%	

*NRDL, National Reimbursement Drug List*.

When not considering subsequent treatment costs (scenario 1), comparing the two market scenarios (without/with geptanolimab in the NRDL), the model estimated that the total annual reimbursement budget would increase by $1,458,842, $1,844,493, and $2,190,023 in 2022, 2023, and 2024, respectively. These increases were mainly driven by the longer period for which patients receiving geptanolimab were progression-free compared with that for patients receiving chidamide.

When considering subsequent treatment costs (scenario 2), there was a shift from cost increases to savings, with the model estimating that the total annual reimbursement budget would decrease by $38,087,822, $64,021,668, and $84,387,363 in 2022, 2023, and 2024, respectively. These decreases were mainly driven by (1) the higher annual disease progression rate in chidamide patients compared with that in geptanolimab patients, and (2) the higher average annual costs of subsequent treatment for chidamide patients compared with that for geptanolimab patients.

**Figure 3:**
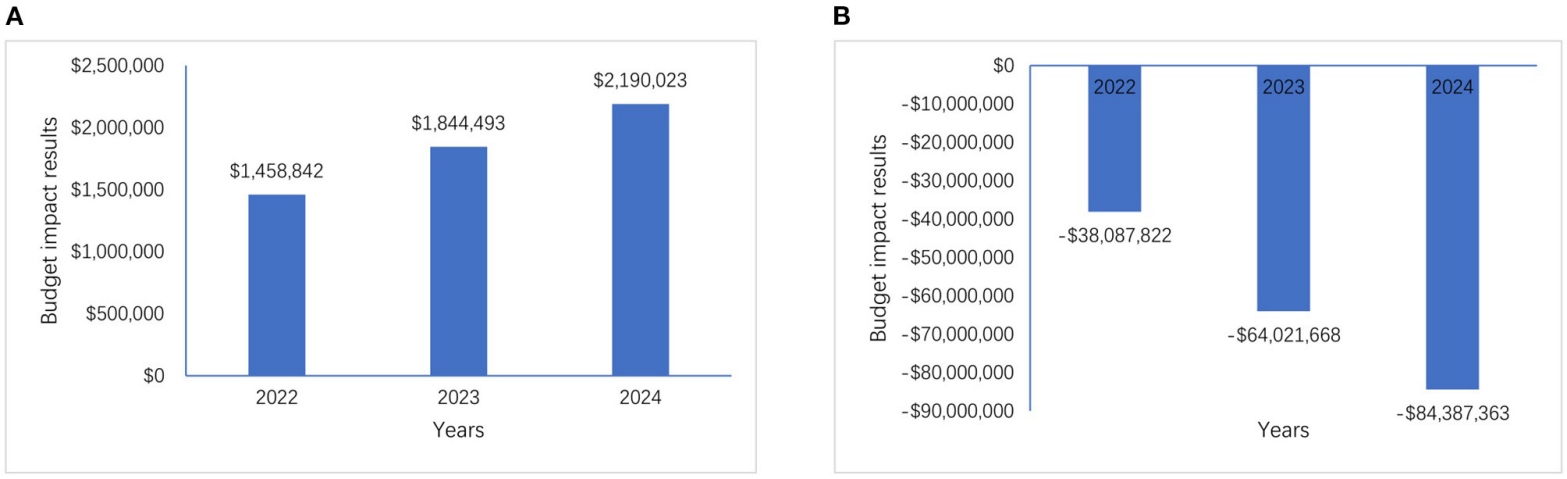
Budget impact results of two cost-scope scenarios, **(A)** is the budget impact results without considering subsequent treatment costs and **(B)** is the budget impact results with considering subsequent treatment costs.

The differences in the budget impact of the two cost-scope scenarios were significant, at $39,546,664, $65,866,161, and $86,577,386 in 2022, 2023 and 2024, respectively. The results of the comparative study are shown in Figure 3 and Appendix 4.

## Discussion

We systematically reviewed BIAs for anticancer drugs conducted over the last 5 years and included 29 studies in our analysis. These studies were conducted in 11 countries and were performed from different perspectives including those of the payer, healthcare system, and society. They were representative of both the increasing number of BIAs for anticancer drugs and the main approaches used in BIAs for anticancer drugs. To the best of our knowledge, changes to the disease spectrum have resulted in increasing numbers of patients being diagnosed with cancer worldwide, leading to more and more innovative anticancer drugs being developed by pharmaceutical companies and approved by various governments (71, 72). Because of the higher prices of these innovative anticancer drugs compared with that of traditional drugs, BIAs have become an important decision-making tool, providing valuable evidence to support decision-making on whether to include these innovative anticancer drugs in reimbursement lists. Thus, the results of BIAs can influence reimbursement decisions and policies. The accuracy and reliability of BIA results in relation to anticancer drugs depend on numerous factors, of which the scope of costs is one of the most important because of the complex treatment procedures and disease progression patterns among cancer patients (73, 74), especially when costs other than those for the target drugs are also covered by the same payer. Thus, we analyzed the scope of costs considered in the BIAs included in our systematic review and compared the budget impact results of two cost-scope scenarios as an example.

### Main Findings

The results of our systematic review revealed that although BIA guidelines from the ISPOR, the NICE, and various countries indicate that costs related to decision-making should be included in BIAs (15, 16, 19–27), most BIAs for anticancer drugs did not include a rational scope of costs. In particular, they tended to ignore the costs of subsequent treatment. This finding is similar to that of a previous systematic review by Han et al. (17), although they only reviewed BIAs for drugs used in the treatment of lung cancer and did not focus on the scope of costs. Nonetheless, more than half of the studies they examined did not consider subsequent treatment costs. This incomplete consideration of the scope of costs may generate inaccurate and unrealistic BIAs. For example, Appukkuttan et al. (42) aimed to compare the budget impact of the use of darolutamide for the treatment of non-metastatic castration-resistant prostate cancer. Although they emphasized that patients were assumed to continue to be treated with the target drugs until disease progression, they did not include subsequent treatment costs after disease progression, which they noted as a limitation of their study.

The results of our comparative study showed that for some anticancer drugs, the inclusion of subsequent treatment costs can result in significant differences in the budget impact, even replacing costs with savings in some cases. There are two main reasons for this: first, innovative anticancer drugs usually have a better treatment effect, albeit over a longer treatment duration at a higher price (72). Thus, if BIAs do not include subsequent treatment costs, the higher costs involved in longer treatment duration at a higher price using a comparator will not be captured. Second, innovative anticancer drugs generally reduce the rate of progression in patients compared with traditional anticancer drugs (75), meaning that patients receiving innovative anticancer drugs require less subsequent treatment. These savings can counteract the additional costs of innovative anticancer drugs used prior to disease progression, sometimes even reversing the impact on the budget, resulting in cost savings for the payer. The example presented in our comparative study illustrates this well. The treatment duration under geptanolimab is longer than that under chidamide, and geptanolimab is more expensive than chidamide. Thus, if we do not consider subsequent treatment, the costs for patients treated with geptanolimab will certainly be higher than those for patients treated with chidamide. However, patients treated with geptanolimab had a lower progression rate and lower average treatment costs for subsequent treatment regimens than those treated with chidamide, resulting in lower overall costs for patients treated with geptanolimab when the costs of subsequent treatment were included.

### Recommendations Regarding BIAs for Anticancer Drugs

BIAs for anticancer drugs need to rationally consider the scope of costs in accordance with BIA guidelines, for instance, the ISPOR good practice guidelines (15, 16), before they are presented to the appropriate budget holder and/or published. Researchers conducting BIAs should divide the costs into three categories: the costs of the drug itself, condition-related costs, and indirect costs. The costs of the drug can be calculated based on the unit price and the estimated treatment duration. Condition-related costs include monitoring and testing costs, administration costs, management costs, and subsequent treatment costs. Our results showed that subsequent treatment costs are significant and need to be considered in BIAs for anticancer drugs. If researchers do not consider subsequent treatment costs in their base-case analysis, they should state the reason for their decision and include these costs in accompanying scenario analyses. However, because of the complexity of the cancer treatment regimen, there is no optimal approach to calculating the subsequent treatment costs for all anticancer-drug BIAs. Researchers can base their calculations on reasonable assumptions (e.g., the proportion of patients in whom the disease progresses based on clinical trials) or real-world data (e.g., the subsequent treatments received by patients). These assumptions and data need to be applied consistently to both the target drug and its comparator(s). Indirect costs should not be considered routinely because these costs are not relevant to the payers and budget holders at most of time. However, they should be considered when they can be predicted and are estimated to be significant. Because in some countries, payers are also responsible for the increased health care costs (e.g., community health care costs) due to rehabilitation and decreased hospital stay of patients.

In addition, it should be noted that although a comprehensive scope of costs is important in BIAs for anticancer drugs, not all BIAs need to consider all related costs. When the target drug and its comparator(s) display no differences in terms of treatment effects and only differ in price, such as biosimilars, researchers do not need to consider a comprehensive scope of costs. Because the consumption of medical resources during subsequent treatment regimens are similar, this will not have a significant effect on the BIA. This is why we excluded BIAs for biosimilars from our systematic review (76–78).

There is another important point that researchers need to ensure the transparency of BIAs for anticancer drugs, because some published BIAs are too simple and do not provide enough cost information, such as detailed parameters and assumptions related to costs (65). These will make decision makers confused about accuracy and reliability of BIA results, and they cannot make decisions based on these evidences. Researchers should (1) clearly describe model structure and its logical relations with costs, (2) state all assumptions of cost calculation and reasons for them, (3) demonstrate all costs parameters with their values, units and sources, and (4) illustrate the calculation methods or formulas used.

### Strengths and Limitations

BIAs and affordability estimates for anticancer drugs, especially innovative drugs, have become increasingly important for reimbursement decision-makers in many countries in recent years. Our study is the first systematic review of BIAs for anticancer drugs and also the first to discuss the scope of costs in relation to BIAs. Among the previous studies, Jahn et al. (79) published a methodological review of BIAs for cancer screening, Abdallah et al. (80) published a methodological quality assessment of BIAs for orphan drugs, and Han et al. (17) published a review of BIAs for antitumor drugs used in the treatment of lung cancer. Although these reviews mentioned the scope of costs, they only provided brief summaries, and did not focus on this aspect. In contrast, in our study, we clearly identify the cost-scope limitations of previous BIAs, reminding researchers and decision-makers of the need to pay more attention to cost-scope issues to improve the accuracy and reliability of their BIAs. In addition, we compared the results of BIAs either considering or ignoring subsequent treatment costs. The results of this comparative study revealed the importance of the scope of costs.

Our systematic review has several limitations. First, BIAs for anticancer drugs are usually undertaken as a supplementary exercise in addition to the CEA that is submitted to budget holders, instead of being prepared independently. Therefore, the BIAs included in our systematic review might only represent a subset of all anticancer-drug BIAs, and some relevant studies may have been overlooked. Second, because the treatment regimens for various types of cancer differ and are generally complex, there is no optimal approach to calculating costs, and thus different approaches may have resulted in different outcomes in terms of budget impact. However, we did not consider this issue in our comparative study. Third, in addition to the scope of costs, there are several other factors influencing the accuracy and reliability of BIAs, such as uncertainty and scenario analyses, validation, and data sources. However, because these were not the focus of our study, we did not consider these factors. Fourth, although nowadays almost each jurisdiction has their own BIA guideline, we only included 10 guidelines as reference. Because some BIA guidelines are not available in public databases or published in other than English. This limitation restricted us from comprehensively reviewing all cost-scope recommendations.

## Conclusions

Most BIAs for anticancer drugs do not rationally consider the scope of costs, which is not in line with the recommendations of the BIA guidelines, and is unrealistic. Our comparative study showed that the difference in budget impact resulting from considering different cost scopes was significant, especially when the same payer was responsible for both the target intervention costs and condition-related costs. Thus, researchers undertaking BIAs for anticancer drugs and reimbursement decision-makers should pay more attention to the scope of costs to improve the rationality, accuracy, reliability, and equity of BIAs for anticancer drugs and the related reimbursement policies.

## Data Availability Statement

The original contributions presented in the study are included in the article/Supplementary Material, further inquiries can be directed to the corresponding author/s.

## Author Contributions

HL: takes responsibility for the data source and the accuracy of the modeling analysis. YM and HL: study design. YM and YL: literature search and data extraction. YM: drafting of manuscript. AM: critical revision of the manuscript. All authors contributed to the article and approved the submitted version.

## Conflict of Interest

The authors declare that the research was conducted in the absence of any commercial or financial relationships that could be construed as a potential conflict of interest.

## Publisher's Note

All claims expressed in this article are solely those of the authors and do not necessarily represent those of their affiliated organizations, or those of the publisher, the editors and the reviewers. Any product that may be evaluated in this article, or claim that may be made by its manufacturer, is not guaranteed or endorsed by the publisher.
