# Supplementary material for: Is the Scope of Costs Considered in Budget Impact Analyses for Anticancer Drugs Rational? A Systematic Review and Comparative Study
Source: Front Public Health. 2021 Nov 5;9:777199. doi: 10.3389/fpubh.2021.777199 (PMC8602071; doi:10.3389/fpubh.2021.777199)
Supplement: Supplementary file 1 [file Table_1.DOCX]

**Appendix list**

# Appendix 1 - Search strategy (searching from 1 January 2016 until 26 July 2021)

| **Database** | **Search Terms** | **Results** | **Total** |
| --- | --- | --- | --- |
| PubMed | #1 (((((budget impact*[Title/Abstract]) OR (budgetary impact*[Title/Abstract])) OR (budget impact analy*[Title/Abstract])) OR (budgetary impact analy*[Title/Abstract])) OR (budget impact stud*[Title/Abstract])) OR (budgetary impact stud*[Title/Abstract]) | 200 | 1367 |
|  | #2 ("financial impact*"[Title/Abstract] OR "economic impact*"[Title/Abstract] OR "economic analy*"[Title/Abstract]) AND "budget*"[Title/Abstract] |  |  |
|  | #3 ((((((((Neoplasia[Title/Abstract]) OR (Neoplasias[Title/Abstract])) OR (Neoplasm[Title/Abstract])) OR (Tumors[Title/Abstract])) OR (Tumor[Title/Abstract])) OR (Cancer[Title/Abstract])) OR (Cancers[Title/Abstract])) OR (Malignancy[Title/Abstract])) OR (Malignancies[Title/Abstract]) |  |  |
|  | #4 (#1 OR #2) AND #3 Filters: in the last 5 years |  |  |
| Web of Science Core Collection | #1 (((((TS=(budget impact*)) OR TS=(budgetary impact*)) OR TS=(budget impact analy*)) OR TS=(budgetary impact analy*)) OR TS=(budget impact stud*)) OR TS=(budgetary impact stud*) | 525 |  |
|  | #2 (((TS=("financial impact*")) OR TS=("economic impact*")) OR TS=("economic analy*")) AND TS=("budget*") |  |  |
|  | #3 ((((((((TS=(Neoplasia)) OR TS=(Neoplasias)) OR TS=(Neoplasm)) OR TS=(Tumors)) OR TS=(Tumor)) OR TS=(Cancer)) OR TS=(Cancers)) OR TS=(Malignancy)) OR TS=(Malignancies) |  |  |
|  | #4 (#1 OR #2) AND #3 Timespan: 2016-01-01 to 2021-07-26 |  |  |
| EMBASE | #1 'budget impact*':ab,ti OR 'budgetary impact*':ab,ti OR 'budget impact analy*':ab,ti OR 'budgetary impact analy*':ab,ti OR 'budget impact stud*':ab,ti OR 'budgetary impact stud*':ab,ti | 590 |  |
|  | #2 ('financial impact*':ab,ti OR 'economic impact*':ab,ti OR 'economic analy*':ab,ti) AND 'budget*':ab,ti |  |  |
|  | #3 neoplasia:ab,ti OR neoplasias:ab,ti OR neoplasm:ab,ti OR tumors:ab,ti OR tumor:ab,ti OR cancer:ab,ti OR cancers:ab,ti OR malignancy:ab,ti OR malignancies:ab,ti |  |  |
|  | #4 ((#1 OR #2) AND #3) AND (2016:py OR 2017:py OR 2018:py OR 2019:py OR 2020:py OR 2021:py) |  |  |
| Cochrane Library | #1 (budget impact*):ti,ab,kw OR (budgetary impact*):ti,ab,kw OR (budget impact analy*):ti,ab,kw OR (budgetary impact analy*):ti,ab,kw OR (budget impact stud*):ti,ab,kw OR (budgetary impact stud*):ti,ab,kw | 52 |  |
|  | #2 (("financial impact*"):ti,ab,kw OR ("economic impact*"):ti,ab,kw OR ("economic analy*"):ti,ab,kw) AND ("budget*"):ti,ab,kw |  |  |
|  | #3 (Neoplasia):ti,ab,kw OR (Neoplasias):ti,ab,kw OR (Neoplasm):ti,ab,kw OR (Tumors):ti,ab,kw OR (Tumor):ti,ab,kw OR (Cancer):ti,ab,kw OR (Cancers):ti,ab,kw OR (Malignancy):ti,ab,kw OR (Malignancies):ti,ab,kw |  |  |
|  | #4 (#1 OR #2) AND #3 with Cochrane Library publication date between 2016-01-01 and 2021-07-26 |  |  |

# Appendix 2: Detailed information of model inputs

**Target interventions related parameters**

| **Parameters** | **Value** | **Sources** |
| --- | --- | --- |
| Patients average weight (kg) | 60.4 | Clinical trials of geptanolimab and chidamide |
| Days per month (d/m) | 30 | Fixed days |
| Days per week (d/w) | 7 |  |
| Months per year (m/y) | 12 |  |
| Patients inhospotal days per month in PFS (d) | 3 | Expert interview |
| Unit price of geptanolimab ($/vial) | 309.40 | Assumption |
| Specifications for geptanolimab (mg/vial) | 70 | Clinical trial of geptanolimab |
| Dose of geptanolimab (mg/kg/2w) | 3 |  |
| Median PFS of geptanolimab patients with R/R PTCL (m) | 3.7 |  |
| Medication cycle of geptanolimab (w) | 2 |  |
| Dosage of geptanolimab per cycle (mg/2w) | 181.2 | Calculation |
| Vials used per cycle of geptanolimab (vials/2w) | 3 |  |
| Annual cycles of geptanolimab (2w) | 8 |  |
| Unit price of chidamide ($/box) | 1273.49 | Clinical trial of chidamide, National medical insurance payment standard,Instructions for chidamide |
| Specifications for chidamide (mg/box) | 120 |  |
| Median PFS of chidamide patients with R/R PTCL (m) | 2.5 |  |
| Medication cycle of chidamide (w) | 1 |  |
| Dosage of chidamide per cycle (mg/w) | 60 | Calculation |
| Vials used per cycle of chidamide (boxes/w) | 0.5 |  |
| Annual cycles of chidamide (w) | 11 |  |

PFS: progression free survival; R/R PTCL: relapsed or refractory peripheral T cell lymphoma

**Inspection and testing related parameters**

| **Items** | **Unit price** | **Frequency (times per year)** | **Proportion of patients** | **Sources** |
| --- | --- | --- | --- | --- |
| Blood biochemistry | 25.06 | 48 | 100% | Unit price of each item is based on the median price of 12 China provinces, data of frequency and proportion is based on expert interview |
| Humoral and cellular immunity | 38.68 | 16 | 100% |  |
| Blood routine examination | 1.47 | 48 | 100% |  |
| Bone marrow aspiration | 11.14 | 4 | 100% |  |
| Bone marrow biopsy | 10.44 | 4 | 100% |  |
| Immunohistochemistry | 15.47 | 4 | 100% |  |
| Bone marrow streaming | 7.74 | 4 | 100% |  |
| FISH | 123.76 | 2 | 100% |  |
| Karyotyping | 23.21 | 2 | 100% |  |
| CT | 21.27 | 24 | 100% |  |
| PET-CT | 618.80 | 8 | 100% |  |
| Ultrasonography | 4.64 | 16 | 100% |  |
| MRI | 67.91 | 8 | 30% |  |
| Pathology consultation | 20.27 | 2 | 100% |  |

**Hospitalization related parameters**

| **In-hospital patient medical resource consumption (except drugs)** |  |  | **Sources** |
| --- | --- | --- | --- |
| Average inhospital days in PFS (d) | Geptanolimab | 11.1 | Calculation |
|  | Chidamide | 7.5 |  |
| Bed ($/d) | | 6.96 | Unit price of each item is based on the median price of 12 China provinces |
| Nursing ($/d) | | 2.01 |  |
| Monitoring ($/d) | | 18.56 |  |

PFS: progression free survival

**III-Ⅳ grade adverse events related parameters**

| **III-Ⅳgrade adverse events** | **Intervention** | **Cost per processing ($)** | **Probability of occurrence in PFS** | **Sources** |
| --- | --- | --- | --- | --- |
| Geptanolimab |  |  |  |  |
| Anemia | Blood transfusion | 34.03 | 1.82% | Unit price of each item is based on the median price of 12 China proviences, Adverse events data is based on clinical trials of geptanolimab and interventions are based on expert interview |
| Platelet count decreased | Platelet raising drugs | 19.89 | 2.74% |  |
| Abnormal liver function | Hepatic protectant | 1.07 | 1.82% |  |
| Upper respiratory tract infection | Oral antibiotics | 0.12 | 1.82% |  |
| Lung infection | Infusion antibiotics | 1.80 | 1.82% |  |
| Autoimmune hemolytic anemia | Blood transfusion+ hormone | 83.23 | 1.82% |  |
| Chidamide |  |  |  |  |
| Platelet count decreased | Platelet raising drugs | 19.89 | 2.12% | Unit price of each item is based on the median price of 12 China proviences, Adverse events data is based on clinical trials of chidamide and interventions are based on expert interview |
| Neutropenia | Leucocyte increasing agent | 25.45 | 0.90% |  |
| Anemia | Blood transfusion | 34.03 | 0.44% |  |
| Lung infection | Infusion antibiotics | 1.80 | 0.17% |  |

**Proportions of each subtype patients receiving each subsequent treatment regimen and regimen costs**

| **Subsequent treatment regimens** | **Patient proportions of subsequent treatment regimens for each subtype of R/R PTCL** | | | | | | | | | | | | **Average costs per cycle for each regimen ($/2w) *** | **Sources** |
| --- | --- | --- | --- | --- | --- | --- | --- | --- | --- | --- | --- | --- | --- | --- |
|  | ENKTL | | PTCL-NOS | | AITL | | ALK+ALCL | | ALK-ALCL | | Other subtypes | |  |  |
|  | Geptanolimab patients | Chidamide patients | Geptanolimab patients | Chidamide patients | Geptanolimab patients | Chidamide patients | Geptanolimab patients | Chidamide patients | Geptanolimab patients | Chidamide patients | Geptanolimab patients | Chidamide patients |  |  |
| Salvage chemotherapy | 60% | 0% | 70% | 70% | 70% | 70% | 80% | 75% | 80% | 75% | 70% | 70% | 843.99 | The proportion of subsequent treatment regimens in different subtypes were obtained from expert interview |
| Salvage chemotherapy+Chidamide | 20% | 15% |  | 0% | 10% | 0% | 10% | 5% | 10% | 5% | 10% | 0% | 2117.48 |  |
| Salvage chemotherapy+PD-1 | 10% | 75% | 10% | 30% | 10% | 30% | 5% | 15% | 5% | 15% | 10% | 30% | 2985.14 |  |
| Chidamide monotherapy | 0% | 0% | 10% | 0% | 10% | 0% | 0% | 0% | 0% | 0% | 10% | 0% | 1273.49 |  |
| PD-1 monotherapy | 0% | 0% | 0% | 0% | 0% | 0% | 0% | 0% | 0% | 0% | 0% | 0% | 2141.15 |  |
| Crizotinib (ALK+ALCL) | 0% | 0% | 0% | 0% | 0% | 0% | 5% | 5% | 5% | 5% | 0% | 0% | 991.07 |  |
| Participating in clinical trials | 10% | 10% | 0% | 0% | 0% | 0% | 0% | 0% | 0% | 0% | 0% | 0% | 0.00 |  |
| Palliative radiotherapy | 10% | 10% | 10% | 10% | 10% | 10% | 10% | 10% | 10% | 10% | 10% | 10% | 4641.00 |  |

R/R PTCL: relapsed or refractory peripheral T cell lymphoma; ENKTL, extraoral natural killer/T cell lymphoma, nasal type; ALCL, anaplastic large-cell lymphoma; ALK, anaplastic lymphoma kinase; NOS, not otherwise specified; AITL: Angioimmunoblastic T-cell lymphoma, * Detailed calculation is shown in the table below

**Detailed costs calculation of each subsequent treatment regimen**

| **Detailed subsequent treatment regimens** | | **Proportion** | **Drugs for each regimen** | **Medication cycle** | **Unit dose (mg/m^2)** | **Days per medication cycle** | **Total dosage per person per medication cycle** | **Treatment pattern** | **Average dose per cycle per person (mg/2w)** | **Specification (mg/ box)** | **Price ($/box) (median price of 12 provinces)** | **Unit Price ($/mg)** | **Average drug costs per cycle for each specific regimen ($/2W)** | **Average drug costs per cycle for each class of regimen ($/2W)** | **Sources** |
| --- | --- | --- | --- | --- | --- | --- | --- | --- | --- | --- | --- | --- | --- | --- | --- |
| Salvage chemotherapy | Bendamustine (2A) | 5% | 120mg/m2, d1, d2 | 21 days | 120 | 2 | 420 | Sustained | 280 | 100 | 649.74 | 6.50 | 1819.27 | 843.99 | The proportions were obtained from expert interview, Unit price of each drug is based on the median price of 12 China provinces |
|  | GDP (2A) | 10% | Gemcitabine 1000mg/m2, d1, d8 | 21days | 1000 | 2 | 3500 | Sustained | 2333 | 1000 | 94.33 | 0.09 | 2369.63 |  |  |
|  |  |  | Cis-platinum 75mg/m2, d1 |  | 75 | 1 | 131.25 | Sustained | 88 | 30 | 4.33 | 0.14 |  |  |  |
|  |  |  | Dexamethasone 40mg, d1-4 |  | 40 | 4 | 280 | Sustained | 187 | 4 | 45.79 | 11.45 |  |  |  |
|  | GemOx (2A) | 40% | Gemcitabine 1000mg/m2, d1 | 14 days | 1000 | 1 | 1750 | Sustained | 1750 | 1000 | 94.33 | 0.09 | 701.11 |  |  |
|  |  |  | Oxaliplatin 100mg/m2, d1 |  | 100 | 1 | 175 | Sustained | 175 | 100 | 306.31 | 3.06 |  |  |  |
|  | ICE (2A) | 25% | Ifosfamide 5g/m2, d2(100% dose of methylene sodium), continuous infusion for 24h | 21 days | 5 | 1 | 8.75 | Sustained | 6 | 1000 | 10.65 | 0.01 | 119.59 |  |  |
|  |  |  | Carboplatin (AUC=5,single dose ≤ 800mg),d2 |  | 800 | 1 | 800 | Sustained | 533 | 100 | 8.34 | 0.08 |  |  |  |
|  |  |  | Etoposide 100mg/m2, d1-3 |  | 100 | 3 | 525 | Sustained | 350 | 50 | 10.72 | 0.21 |  |  |  |
|  | SMILE | 15% | methotrexate 2g/m2, continuous infusion for 6h, d1 | 28 days | 2 | 1 | 3.5 | Sustained | 2 | 1000 | 27.06 | 0.03 | 1082.15 |  |  |
|  |  |  | calcium folinate 15mg×4, d2-4 |  | 60 | 3 | 315 | Sustained | 158 | 150 | 7.91 | 0.05 |  |  |  |
|  |  |  | Ifosfamide 1500mg/m2, d2-4 |  | 1500 | 3 | 7875 | Sustained | 3938 | 500 | 6.34 | 0.01 |  |  |  |
|  |  |  | Mesna 300mg/m2 ×3, d2-4 |  | 900 | 3 | 4725 | Sustained | 2363 | 400 | 3.48 | 0.01 |  |  |  |
|  |  |  | Dexamethasone 40mg, d2-4 |  | 40 | 3 | 210 | Sustained | 105 | 2.5 | 20.65 | 8.26 |  |  |  |
|  |  |  | Etoposide 100mg/m2, d2-4 |  | 100 | 3 | 525 | Sustained | 263 | 50 | 10.72 | 0.21 |  |  |  |
|  |  |  | L-Asparaginase 6000U/m2, d8, d10, d12, d14, d16, d18, d20 |  | 6000 | 7 | 73500 | Sustained | 36750 | 5000 | 10.83 | 0.00 |  |  |  |
|  | P-GemOx | 5% | Pegaspargase 2500IU/m2, d1 | 21 days | 2500 | 1 | 4375 | Sustained | 2917 | 1500 | 228.60 | 0.15 | 867.95 |  |  |
|  |  |  | Gemcitabine 1250mg/m2, d1 |  | 1250 | 1 | 2187.5 | Sustained | 1458 | 1000 | 94.33 | 0.09 |  |  |  |
|  |  |  | Oxaliplatin 80mg/m2, d1 |  | 80 | 1 | 140 | Sustained | 93 | 100 | 306.31 | 3.06 |  |  |  |
| PD-1 | Sintilimab (2A) | 50% | 200mg, d1 | 21 days | 200 | 1 | 200 | Sustained | 133 | 100 | 2771.91 | 27.72 | 3695.89 | 2141.15 |  |
|  | Pembrolizumab (2A) | 50% | 200mg, d1 |  | 200 | 1 | 200 | Sustained | 133 | 100 | 439.81 | 4.40 | 586.42 |  |  |
| Chidamide |  |  | 30mg, oral, 2 times per week | Sustained | 30 | 2 | 120 | Sustained | 120 | 5 | 53.06 | 10.61 | 1273.49 | 1273.49 |  |
| Crizotinib |  |  | 250mg, oral, 2 times per week | Sustained | 250 | 14 | 7000 | Sustained | 7000 | 250 | 35.40 | 0.14 | 991.07 | 991.07 |  |
| Palliative radiotherapy |  |  | 5 times per 35 days | 35 days |  | 25 | 25 | Sustained | 10 |  | 464.10 | 464.10 | 4641.00 | 4641.00 |  |

# Appendix 3 - Characteristics of included studies (supplementary)

| **First Author** | **Year** | **Title** | **Country** | **Results of budget impact analysis** | **Uncertainty and Scenario Analyses** | **Validation** | **Data sources** |  |
| --- | --- | --- | --- | --- | --- | --- | --- | --- |
|  |  |  |  |  |  |  |  |  |
| Appukkuttan [42] | 2020 | Budget impact analysis of darolutamide for treatment of nonmetastatic castration-resistant prostate cancer | US | Year1-year2-year3-year4-year5: $158,640-$200,539-$150,697-$28,901-−$149,240 | One-way | No | SEER cancer statistics; US Census Bureau; Market research data (Bayer); Published literature; Online interview; Medicare Physician Fee Schedule of the Resource-Based Relative Value Scale; Clinical trial data; Healthcare Cost and Utilization Project; US Bureau of Labor Statistics |  |
| Cai [43] | 2020 | Budget impact of capmatinib for adults with metastatic non-small cell lung cancer harboring a MET exon 14 skipping mutation in the United States | US | Commercial: year1-year2-year3: $9,695-$38,783-$67,725; Medicare: $141,350-$564,706-$985,695 | One-way | No | Published literature; market research data (Novartis); CMS; 2020 CMS Physician Fee Schedule; Red Book wholesale acquisition cost (WAC); 2020 CMS Average Sales Price Drug Pricing file; US Flatiron database; GEOMETRY trial; CMS Clinical Laboratory Fee Schedule; CMS Physician Fee Schedule; Healthcare Cost and Utilization Project national inpatient sample |  |
| Mason [44] | 2021 | Budget Impact of Adaptive Abiraterone Therapy for Castration-Resistant Prostate Cancer | US | -$67,689 | / | No | Publicly available national databases |  |
| Stargardter [45] | 2021 | Budget impact of tepotinib in the treatment of adult patients with metastatic non-small cell lung cancer harboring METex14 skipping alterations in the United States | US | Year1-year2-year3: –$103,337, –$313,790, -$275,414 | One-way | No | Published literatures; National Cancer Institute; Program America’s Health Insurance Plans; Demographics Report; Market research; IBM Micromedex Red Book; CMS; Healthcare Cost and Utilization Project (HCUPnet) |  |
| Wallace [46] | 2020 | The Budget Impact of Including Rucaparib on a US Payer Formulary  for the Treatment of Patients with Metastatic Ovarian Cancer | US | Maintenance: Year1-year2-year3: $3358-$3693-$4029 Treatment: Year1-year2-year3: $6982-$6982-$6982 | One-way | No | Published literatures; US Census Bureau. Current Population Survey; Medicare Clinical Laboratory Fee Schedule; CMS Physicians Fee Schedule; Medicare Fee Schedule; SEER; US National Health Expenditures Price index |  |
| Monirul [47] | 2020 | Budget Impact Analysis of Fixed Dose Versus Weight-Based Dosing Regimen of Nivolumab and Pembrolizumab in the Treatment of Non-Small Cell Lung Cancer | France | Additional treatment cost PPPY **Nivolumab:** €3239 **Pembrolizumab:**  1st Line: €18,039 2nd Line: €8418 | One-way | No | Scan Santé platform; ORBIS® patient file; Ar-Kdos® RCP files; Avicenne Hospital; Published literature; French Health Insurance |  |
| Schultz [48] | 2020 | Budget Impact of Enzalutamide for Nonmetastatic Castration-Resistant Prostate Cancer | US | Year1-year3: $106,074-$632,729 | One-way | No | Published literature; RED BOOK; CMS fee schedules; CMS clinical laboratory and physician schedules; Bureau of Labor Statistics Consumer Price Index |  |
| Yamazaki [49] | 2020 | Budget impact analysis of treatment-free remission in nilotinib-treated Japanese chronic myeloid leukemia patients | Japan | Year1-year2-year3(Japanese￥):2,577,451,775- 2,589,441,684-2,458,281,181 | One-way | No | Published literature; Japanese National Health Insurance fee schedule |  |
| Kongnakorn [50] | 2019 | Budget Impact of Including Avelumab As A Second-Line Treatment for Locally Advanced or Metastatic Urothelial Cancer in The United States: Commercial and Medicare Payer Perspectives | US | Commercial: Year1-year2-year3: $31,556-$23,523-$18,360 Medicare: Year1-year2-year3: $309,085-$230,409-$179,831 | One-way | No | Published literature; the US-based SEER dataset; CPI from the Bureau of Labor Statistics; AWP from Red Book for the commercial perspective; ASP from the CMS for the Medicare perspective; the US Oncology Network electronic health records database; Healthcare Blue Book database; CMS; Healthcare Cost and Utilization Project (HCUP) database |  |
| Neeser [51] | 2019 | Budget impact of niraparib as maintenance treatment in recurrent ovarian cancer following platinum-based chemotherapy | US | Population: Year1-year2-year3: -$78,721, -$276,671, -$353,585 Medicare: Year1-year2-year3: -$293,723, -$1,009,729, -$1,289,712 | One-way | No | Published literature; Current Procedural Terminology codes |  |
| Stellato [52] | 2019 | Budget Impact of Dabrafenib and Trametinib in Combination as  Adjuvant Treatment of BRAF V600E/K Mutation-Positive Melanoma  from a US Commercial Payer Perspective | US | Relapse-free survival: Year1-year2-year3: $173,577-$301,323-$206,753 Locoregional recurrence: Year1-year2-year3: $1,656-$5,337-$10,530 Total: Year1-year2-year3: $165,841-$253,376-$129,389 | One-way | No | COMBI-AD trial; Published literature; SEER; data on file, Novartis |  |
| Wu [53] | 2019 | Budget impact analysis of niraparib and olaparib for maintenance treatment of platinum-sensitive, recurrent ovarian cancer in the US | US | Total incremental cost per patient per year Niraparib: $98,174 Olaparib: $90,575 | One-way | No | Clinical trials; Red Book; Published literature; SEER Cancer Statistics Review |  |
| Bly [54] | 2018 | The Budget Impact of Including Necitumumab on the Formulary for First-Line Treatment of Metastatic Squamous Non-Small Cell Lung  Cancer: US Commercial Payer and Medicare Perspectives | US | Year1-year2-year3: $88,394-$237,043-$304,079 | One-way | No | RED BOOK Online database; Published literature; CPI |  |
| Graham [55] | 2018 | Budget Impact Analysis of Afatinib for First-Line Treatment of Patients with Metastatic Non-Small Cell Lung Cancer with Epidermal Growth Factor Receptor Exon 19 Deletions or Exon 21 Substitution Mutations in a US Health Plan | US | Year1-year2-year3-year4-year5: $1,606-$65,542-$140,564-$209,272-$303,368 | One-way | No | Published literature; data on file, Boehringer Ingelheim; Essential Resource-Based Relative Value Scale (RBRVS); Micromedex Red Book online; CPI |  |
| Mistry [56] | 2018 | Budget impact of including ribociclib in combination with letrozole on US payer formulary: first-line treatment of post-menopausal women with HR+/HER2- advanced or metastatic breast cancer | US | Year1-year2-year3: -$125K, -$1036K, -$1850K | One-way | No | Published literature; Novartis, data on file; CPI; 2016 Medicare Physician Fee Schedule |  |
| Goldstein [57] | 2017 | A Pharmacoeconomic Analysis of Personalized Dosing vs Fixed Dosing of Pembrolizumab in Firstline PD-L1-Positive Non–Small Cell Lung Cancer | US | -$825,630,583 | One-way | No | SEER; Published literature; ASP for pembrolizumab |  |
| Bloudek [58] | 2016 | Estimating the Economic Impact of Adding Panobinostat to a US Formulary for Relapsed and/or Refractory Multiple Myeloma: A Budget Impact and Cost-Benefit Model | US | Commercial: -$46,450 Medicare: -$342,169 | One-way | No | 2012 US Census data; Medicare demographic data; SEER database, and published literature; RED BOOK |  |
| Bui [59] | 2016 | Budget Impact of Enzalutamide for Chemotherapy-Naïve Metastatic Castration-Resistant Prostate Cancer | US | $510,641 | One-way | No | SEER database; RED BOOK; CMS ASP pricing files; CMS physician fee schedule; Agency for Healthcare Research and Quality Healthcare Cost and Utilization Project; Published literature |  |
| Silva [60] | 2021 | The Budget Impact of Monoclonal Antibodies Used to Treat Metastatic Colorectal Cancer in Minas Gerais, Brazil | Brazil | Reference scenario CT only Alternative scenario A :BEVA + CT Alternative scenario B :CETUX + CT Alternative scenario C PANIT + CT Scenario A vs Reference scenario:Year1-year2-year3-year4-year5:$ 21,131,270-$ 21,820,930-$ 22,494,830-$ 22,797,420-$ 23,090,440 Scenario B vs Reference scenario:Year1-year2-year3-year4-year5:$ 22,434,110-$ 22,638,760-$22,835,400-$ 22,901,810-$ 22,962,790 Scenario C vs Reference scenario:Year1-year2-year3-year4-year5:$19,946,510-$ 20,389,410-$ 20,820,410-$ 21,010,850-$ 21,193,540 Scenario B vs scenario A:Year1-year2-year3-year4-year5:$ 1,302,840-$ 817,830-$ 340,570-$104,650-$ -127 Scenario C vs scenario A:Year1-year2-year3-year4-year5:$ −1,184,750-$ −1,431,520-$ −1,674,160-$−1,786,560-$ −1,896,900 Scenario C vs scenario B:Year1-year2-year3-year4-year5:$−2,487,600-$−2,249,350-$−2,014,730-$ −1,891,210-$ −1,769,510 | One-way | No | Published literatures; Brazilian Ministry of Health’s National Health Surveillance Agency (ANVISA);National Database of Health dataset ;Brazilian Institute of Geography and Statistics (IBGE); Management System of Procedures; Medicines and OPM (orthosis, prosthesis and medical materials) of SUS Procedure Table Management System (SIGTAP); Integrated Pharmaceutical Assistance Management System; Integrated Direct Administration System; MG Center for Assistance to the Judicialization of Health (NAJS); Public Acta of the MG Purchasing Portal Price Record |  |
| Elsamany [61] | 2021 | Budget impact analysis of subcutaneous (SC) trastuzumab compared to intravenous (IV) trastuzumab in Saudi HER2-positive breast cancer patients | Saudi Arabia | 1st Scenario: Gradual Replacement: IV Trastuzumab: SAR 177,773,234  SC Trastuzumab: SAR 143,245,888 2nd Scenario: Total Replacement: IV Trastuzumab: SAR 177,773,234  SC Trastuzumab: SAR 108,718,542 | One-way | No | Chemotherapy unit and clinical pharmacy records, King Abdullah Medical City |  |
| Westerink [62] | 2020 | Budget impact of sequential treatment with first‑line afatinib versus first‑line osimertinib in non‑small‑cell lung cancer patients  with common EGFR mutations | Dutch | Year1-year2-year3-year4-year5(million): €6.06-€12.25-€12.26-€9.87-€7.72 | One-way | No | Published literatures |  |
| Delgado-Ortega [63] | 2018 | Economic impact of olaparib on maintenance treatment of patients with BRCA-mutation positive, platinum-sensitive relapsing high-grade serous epithelial ovarian cancer in Spain | Spain | Year1-year2-year3-year4-year5: €1,664,662-€3,134,588-€5,278,927-€5,483,913-€5,422,632 | One-way | No | GLOBOCAN database; Published literatures; OCEANS study |  |
| Flannery [64] | 2017 | Budgetary Impact of Cabazitaxel Use After Docetaxel Treatment for Metastatic Castration-Resistant Prostate Cancer(mCRPC) | US | A. Excluding Out-of-Pocket Costs: -$49,546 B. Including All Costs Paid by Plan: -$86,136 | One-way | No | Market research data; CMS July 2015 ASP Pricing File; CPI; Actual utilization rates as of January 2015; unpublished data, IMS Health, 2015 |  |
| Norum [65] | 2017 | Pembrolizumab as second-line therapy in non-small cell lung cancer in northern Norway: budget impact and expected gain—a model-based analysis | Norway | € 5,016,191 | One-way | No | Department of Pathology at the University Hospital of North Norway (UNN) and Nordland Hospital (NH); 2016 price list of the Norwegian Health Economics Administration (NHEA); Hospital Pharmacy of North Norway; Norwegian Medicines Agency (NMA); data from Statistics Norway (www. ssb. no) |  |
| Ortendahl [66] | 2017 | Budget impact of somatostatin analogs as treatment for metastatic gastroenteropancreatic neuroendocrine tumors in US hospitals | US | $−488,615 | One-way | No | Published literature; pricing databases; market share data |  |
| Kulthanachairojana [67] | 2020 | Home-based chemotherapy for stage III colon cancer patients in Thailand: Cost-utility and budget impact analyses | Thailand | $−1,513.37 | DSA: One-way PSA (Probabilistic sensitivity analysis): Monte Carlo simulations | No | National Comprehensive Cancer Network (NCCN); published literature; Drug and Medical Supply Information Center (DMSIC) website |  |
| Hanna [68] | 2021 | Three versus six months of adjuvant chemotherapy for colorectal cancer: A multi-country cost-effectiveness and budget impact analysis | Australia, Denmark, New Zealand, Spain, Sweden and the UK | cost savings over 5 years (USD millions)  Australia:24.7  Denmark:7.4 New Zealand:3.6 Spain:44.4 Sweden:10.2  United Kingdom:61.4 | One-way | No | Published literature; WHO Choice project; Scottish Information Services Division (ISD) unit costs (2019) |  |
| Mennini [69] | 2019 | Rationale and budget impact of bimonthly use of Cetuximab in patients with recurrent and/or metastatic head and neck cancer | Italy | Base case vs EOW 50% scenario,2 months: −€347,063 Base case vs EOW 100% scenario, 2 months: −€694,126 | One-way | No | Published literature; Italian Association of Cancer Registries |  |
| Mennini [70] | 2019 | Budget impact of bimonthly use of cetuximab in patients diagnosed with metastatic colorectal cancer | Italy | Base case vs EOW 50% scenario,10 months results: -€4,889,979 Base case vs EOW 100% scenario, 10 months results: -€15,595,409 | One-way | No | Published literature; Italian Association of Cancer Registries |  |

PPPY: Per year and per patient; SAR: Saudi Riyal; EOW: every-other-week; SEER: Surveillance, Epidemiology, and End Results; CMS: Centers for Medicare & Medicaid Services; AWP: Average wholesale price; ASP: Average sales price; CPI: Consumer Price Index

# Appendix 4-Budget impact results of two cost-scope scenarios

| **Expenditure of payer ($)** | | **Years** | | |
| --- | --- | --- | --- | --- |
|  |  | **2022** | **2023** | **2024** |
| Cost-scope scenario 1 |  |  |  |  |
|  | Without geptanolima NRDL entry | 35,406,592 | 40,637,876 | 44,980,067 |
|  | With geptanolima NRDL entry | 36,865,434 | 42,482,370 | 47,170,090 |
|  | Budget impact results | 1,458,842 | 1,844,493 | 2,190,023 |
| Cost-scope scenario 2 |  |  |  |  |
|  | Without geptanolima NRDL entry | 266,386,588 | 305,744,911 | 338,414,008 |
|  | With geptanolima NRDL entry | 228,298,766 | 241,723,243 | 254,026,645 |
|  | Budget impact results | -38,087,822 | -64,021,668 | -84,387,363 |
|  |  |  |  |  |
| Absolute difference |  | 39,546,664 | 65,866,161 | 86,577,386 |

NRDL: National Reimbursement Drug List
